# A Reduction in Delay Discounting by Using Episodic Future Imagination and the Association with Episodic Memory Capacity

**DOI:** 10.3389/fnhum.2016.00663

**Published:** 2017-01-05

**Authors:** Xiaochen Hu, Helena Kleinschmidt, Jason A. Martin, Ying Han, Manuela Thelen, Dix Meiberth, Frank Jessen, Bernd Weber

**Affiliations:** ^1^Department of Psychiatry and Psychotherapy, Medical Faculty, University of CologneCologne, Germany; ^2^Department of Psychiatry and Psychotherapy, University Hospital of BonnBonn, Germany; ^3^Department of Radiology, University Hospital of BonnBonn, Germany; ^4^Department of Neurology, XuanWu Hospital of Capital Medical UniversityBeijing, China; ^5^German Center for Neurodegenerative DiseasesBonn, Germany; ^6^Department of Epileptology, University Hospital of BonnBonn, Germany; ^7^Center for Economics and Neuroscience, University of BonnBonn, Germany

**Keywords:** delay discounting, episodic future imagination, episodic memory capacity, subjective value, anterior cingulate cortex, left inferior parietal cortex

## Abstract

Delay discounting (DD) refers to the phenomenon that individuals discount future consequences. Previous studies showed that future imagination reduces DD, which was mediated by functional connectivity between medial prefrontal valuation areas and a key region for episodic memory (hippocampus). Future imagination involves an initial period of construction and a later period of elaboration, with the more elaborative latter period recruiting more cortical regions. This study examined whether elaborative future imagination modulated DD, and if so, what are the underlying neural substrates. It was assumed that cortical areas contribute to the modulation effect during the later period of imagination. Since future imagination is supported by episodic memory capacity, we additionally hypothesize that the neural network underlying the modulation effect is related to individual episodic memory capacity. Twenty-two subjects received an extensive interview on personal future events, followed by an fMRI DD experiment with and without the need to perform elaborative future imagination simultaneously. Subjects' episodic memory capacity was also assessed. Behavioral results replicate previous findings of a reduced discount rate in the DD plus imagination condition compared to the DD only condition. The behavioral effect positively correlated with: (i) subjective value signal changes in midline brain structures during the initial imagination period; and (ii) signal changes in left prefrontoparietal areas during the later imagination period. Generalized psychophysiological interaction (gPPI) analyses reveal positive correlations between the behavioral effect and functional connectivity among the following areas: right anterior cingulate cortex (ACC) and left hippocampus; left inferior parietal cortex (IPC) and left hippocampus; and left IPC and bilateral occipital cortices. These changes in functional connectivity are also associated with episodic memory capacity. A hierarchical multiple regression indicates that the model with both the valuation related signal changes in the right ACC and the imagination related signal changes in the left IPC best predicts the reduction in DD. This study illustrates interactions between the left hippocampus and multiple cortical regions underlying the modulation effect of elaborative episodic future imagination, demonstrating, for the first time, empirical support for a relation to individual episodic memory capacity.

## Introduction

Delay discounting (DD) refers to the phenomenon that most people tend to discount future consequences when facing the intertemporal choice between a sooner smaller reward and a later larger reward (Ainslie, 1975; Frederick et al., 2002). This phenomenon impacts a large variety of everyday decisions, related to health, finance and education. Individual differences in DD have been shown to relate to health-related behavior such as substance abuse (Kirby et al., 1999), gambling (Dixon et al., 2003), or overeating (Weller et al., 2008).

A growing body of evidence has considered DD as a target for interventional therapies, in the hope for the treatment of diseases through changes of discount rates (Koffarnus et al., 2013). Behavioral modulations such as future imagination have been shown to reduce individual discount rates in both healthy young subjects and patients (Ungemach et al., 2011; Cheng et al., 2012; Daniel et al., 2013; Lin and Epstein, 2014; Dassen et al., 2016). Neuroimaging studies addressing the modulation effect through future imagination have revealed an association between DD reductions and increased brain activity in medial prefrontal valuation areas (Peters and Büchel, 2010; Benoit et al., 2011). Activation in the medial prefrontal cortex (mPFC) was found to interact with the hippocampus (Peters and Büchel, 2010; Benoit et al., 2011), a key region for episodic memory and episodic future imagination (Schacter and Addis, 2009). These reports suggest a hippocampal-mPFC pathway for this modulation effect: future imagination may increase the functional coupling between the hippocampus and the mPFC, changing the reward valuation process, and altering the representation of future rewards such that it may result in more future oriented choices.

Episodic memory has been conceived as a system that enables people to recollect past experiences. Early lesion study showed that patients with damage within the episodic memory system, including the medial temporal lobe (MTL), had deficits in future imagination (Tulving, 2002). Later, a ground-breaking fMRI study demonstrated that both remembering the past and imagining the future rely on the same brain network (Addis et al., 2007). Episodic memory is now considered a constructive system that enables both the recollection of past events and the simulation of future events (Schacter and Addis, 2007; Schacter et al., 2007, 2012). It has also been demonstrated that individual differences in episodic memory capacity significantly impact the ability of episodic future imagination (Addis et al., 2008; Gamboz et al., 2010). The hippocampus, a key region for both past recollection and future imagination (Addis et al., 2011; Addis and Schacter, 2011), has been suggested to support decision making processes through the interaction with cortical areas via multiple processing pathways (Buckner, 2010). The aforementioned neuroimaging studies (Peters and Büchel, 2010; Benoit et al., 2011) were seminal to demonstrate the hippocampal-mPFC pathway underlying the modulatory effect of future imagination on intertemporal choices. However, it is currently unclear whether additional hippocampal-cortical pathways exist. It is also not clear whether individual differences in this modulation effect arise from differences in episodic memory capacity. Given the relationship between episodic future imagination and individual episodic memory capacity, we assume that neuronal pathways underlying the modulation effect are related to the individual episodic memory capacity.

Episodic future imagination can be divided into an initial retrieval period and a more effortful later elaboration period with both utilizing different neuronal networks. Initial retrieval is characterized by reactivating distributed memory traces, while the later elaboration period is characterized by maintaining the event in the mind and adding more details (Daselaar et al., 2008). Episodic future imagination typically activates a neural network that includes the mPFC, posterior cingulate cortex (PCC), retrosplenial cortex, MTL (incl. hippocampus) and lateral parietal cortex (Schacter et al., 2012). The later elaboration period of future imagination additionally activates cortical areas such as lateral prefrontal and parietal cortices plus the precuneus (Addis et al., 2007). A lesion study (Berryhill et al., 2010) showed that patients with damage in prefrontal and parietal cortical regions were selectively impaired in the elaboration of the constructed events, but not the construction *per se*. These results indicated that the lateral prefrontal and parietal cortical regions have a unique contribution to the elaboration of the episodic future imagination. Earlier fMRI studies on the modulation effect of future imagination on intertemporal choices did not separately investigate the influences of different processing stages of imagination (Peters and Büchel, 2010; Benoit et al., 2011). In the present study, we investigate the neural processes that underlie the modulation effect on DD by using more elaborated future imagination and its specific relation to individual differences in episodic memory capacity.

Our paradigm is an adaptation of the Peters and Büchel (2010) paradigm (P&B paradigm). We made the following paradigm adjustments to promote the elaboration of future events: (1) a detailed pre-scan interview was implemented for the construction of events that fulfills the need to have a specific time and place (Levine et al., 2002) (P&B paradigm: asked subjects to compile a list of future event without an extensive interview); (2) We gave explicit instructions during the fMRI task to be elaborative (i.e., use your imagination to be as vivid as possible) (P&B paradigm: subjects were not instructed to use mental imagery); (3) We used a longer imagination period (8 s) during the fMRI task to give time for elaborative imagery (P&B paradigm: used a 3 s period). During the analysis, we model the neural activity separately for the initial period (0–3 s) and the later period (3–8 s). Separating the imagination period in this way allows us to direct compare our results with the early study (Peters and Büchel, 2010) whilst additionally assess the influence of a longer imagination period. Since all subjects were able to retrieve their personal event immediately after seeing the cue word, these two periods closely resemble the construction and elaboration period of imagination. We assume that episodic future imagination reduces individual discount rates (behavioral modulation effect) and that more cortical areas are engaged in the later period of future imagination. Following the suggestion that the hippocampal region may support decision-making through an interaction with cortical areas via multiple pathways (Buckner, 2010), we hypothesize that the hippocampus functionally interacts with multiple cortical areas during prolonged and elaborated future imagination by interacting with more cortical regions in the later period of imagination.

## Materials and methods

### Subjects

Twenty-two right-handed subjects (14 female) took part in the study (age: 24 ± 3 years, range: 19–28 years). All subjects reported to be in “good health, with no history of psychiatric or neurological disorders” and did not take any medication. All subjects gave written informed consent. The study was approved by the local ethics committee (University of Bonn, Germany) in accordance with the Declaration of Helsinki.

### Experimental procedure

Our main experiment (the fMRI DD experiment) was individually tailored with regard to the future reward values and the future events. Therefore, additional tasks were conducted prior to the fMRI experiment to acquire individual choice preferences and personalized future events. This whole study took place in 2 days.

During the first day, subjects were asked to perform a behavioral DD experiment to estimate the individual discount parameters (Section Behavioral DD Pretest). Next, subjects performed a verbal list learning task to obtain their episodic memory capacity (Section Verbal List Learning Task). Finally, subjects were given instructions to help them prepare their personalized future events at home (Section Preparation for Future Imagination).

During the second day, a pre-scan interview to assess the subjects' personal episodic future events was undertaken, and a corresponding cue word generated for each future event (Section Pre-scan Interview). These cue words were used in the DD fMRI task to cue subjects to retrieve their personalized future event. Immediately after the pre-scan interview, subjects were trained on the fMRI DD task using a laptop computer outside the scanner. Subsequently, subjects performed the fMRI DD task that contained two sessions (Section fMRI DD Task with and without Future Imagination). Finally, a brief post-scan interview was conducted to evaluate the subjects' imagination (Section Post-scan Interview).

#### Behavioral DD pretest

On the first day, all subjects performed a computer-based behavioral DD experiment, in which subjects were asked to repeatedly make a choice between a 20 € immediate reward and a larger future reward (range 21–200 €) for six delay intervals (i.e., 1, 2 weeks, 1, 3, 6 months, and 1 year). Subjects were given no response time limit. Future rewards systematically varied across trials guided by a random adjustment procedure (Richards et al., 1999).

The DD pretest choice data was then used to estimate individual choice preferences. For each delay period, an indifferent point (IP) was calculated using a binary logistic regression, performed in SPSS (Version 22, IBM Corp., NY). For each logistic regression analysis, the value of future reward was entered as an independent variable and the subjects' response as the dependent variable. The IP value was estimated from the logistic regression, which corresponded to the future reward value at which the subject was indifferent between the immediate and the delayed reward. The IP values were used for the individual adjustment of the future reward value in the fMRI DD task, so that in approximately half of the trials, the future options were chosen by our participants.

#### Verbal list learning task

On the first day, all subjects performed a verbal list learning task to estimate their episodic memory capacity. The German version of the Verbal Learning and Memory Test (VLMT) (Helmstaedter and Durwen, 1990) was used, an equivalent to the well-known Auditory Verbal Learning Test (AVLT) (Rey, 1964; Lezak, 1983). Subjects were asked to listen to a list of 15 words, and then to recall as many words as possible. The learning of this word list was repeated five times (learning trials 1–5). Next, subjects were asked to listen to and repeat a second list of 15 words (interference trial). Subsequently, they were asked to recall words from the first list (immediate recall). And then they were asked once again, half an hour later, to recall the first word list (delayed recall). The scores on the first learning trial and the sum of five learning trials were derived and included in the statistical analyses of the current study. We did not analyze the latter two trials (immediate recall, delayed recall) since most subjects reached the ceiling effect.

#### Preparation for future imagination

At the end of the first day, subjects were instructed to prepare at home for an interview that took place on the second day. As preparation, subjects were asked to think about two individual future events for each of the delay intervals (i.e., 1, 2 weeks, 1, 3, 6 months, and 1 year), and to generate a verbal cue for each event, so that in total they had 12 future events. Subjects were instructed that the future events should have a specific time and place, and should not evoke negative feelings. The future event should also be realistic and have personal relevance. Subjects were asked to think about the details of the events, such as what they could see, hear, smell, think, and feel during the imagination.

#### Pre-scan interview

On the second day, a structured interview on personalized episodic future events was conducted. The interview was modified from an autobiographical interview method (Levine et al., 2002), in which subjects were asked to spontaneously describe their events as detailed as possible. If subjects' spontaneous description did not fulfill the requirement of specificity (e.g., no information about time or place), general probes were used to encourage greater recall of details (i.e., “Can you tell me a specific instance of …?”). If description still lacked time/place information, specific questions were raised to elicit additional details (i.e., “Could you see any landmarks in the surroundings?”). During the interview, we also checked that all future events reported were realistically possible for our subjects in the future. At the end of the interview, to verify each subject's response, all verbal cues were read aloud to the subject to ensure that constructed events were retrieved immediately. All subjects reported success. Subjects were asked to rate the difficulty, valence and arousal of each imagined future event on a point scale from 1 to 9, with 9 indicating extremely difficulty, positive valence and high arousal and 1 indicating extremely easy, negative valence and low arousal.

#### fMRI DD task with and without future imagination

On the second day, subjects completed a practice session of the fMRI task on a laptop computer outside the scanner immediately after the pre-scan interview and prior to the actual fMRI experiment, in order to familiarize with the procedure. All subjects have reported that it was not difficult to conduct the task.

**Figure 1:**
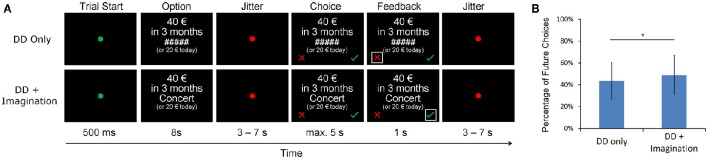
**(A)** fMRI delay discounting (DD) experiment design. Each trial started with a green fixation point and followed by a choice question for 8 s. In the DD + imagination condition, the options were coupled with verbal cues of future events, while not in the DD only condition. In both conditions, subjects were instructed to make intertemporal choices between the fixed immediate reward (20 €) and a later larger reward (>20 €). They should press the green check for accepting the future reward, and press the red cross for refusing the future reward. In the DD + imagination condition, subjects were instructed to additionally imagine their future events as detailed as possible during the intertemporal choices. **(B)** Percentage of future choices under both the DD only condition and the DD + imagination condition; ^*^p < 0.05.

The fMRI task (Figure 1A) had an event-related design and consisted of two sessions with 48 trials each. In each session, 24 trials were randomly assigned to the DD only condition, and the other trials were assigned to the DD + imagination condition. All trials started with a green fixation point (0.5 s), after which an intertemporal choice question with a fixed amount of the immediate reward option (20 € today) and a future reward option (>20 €) was presented (8 s). In the DD only condition, a chain of hashtag signs (“#####”) was presented between the two options. In the DD + imagination condition, a cue word of a personalized future event was presented between the two options. In both conditions, subjects were instructed to think about which option they prefer (but not to press the button immediately). In the DD + imagination condition, subjects were required to additionally retrieve their future events according the cue word, and to elaborate the events as detailed as possible. Then, a jittered period was presented (3–7 s). Subsequently, the subjects were asked to press the respective response button as soon as possible (choice period; maximum 5 s). Subjects were instructed to press the right button (the green check) if they accepted future reward, and to press the left button (the red cross) if they refuse it. Finally, a feedback of their choice was given (1 s). For each delay period, eight different future reward amounts were calculated based on the estimated IP value from the behavioral DD pretest using the equation: IP_n_ + (IP_n_ − IP_n−1_) ^*^ x, in which IP_n_ was the IP of that delay period, IP_n−1_ was the IP of the last delay period, and x varied from −1.05 to 1.05 with an interval of 0.3.

#### Post-scan interview

After the fMRI session, a post-scan interview was conducted. Subjects were asked about their imagined events from the scanning session. We confirmed that all subjects imagined the same events during fMRI sessions as they reported in the pre-scan interview. Subjects were then required to rate the difficulty, valence and arousal of each imagined future event on a 9-point scale. The reward from the fMRI task has been realized by drawing lots after the fMRI session. Independently, subjects received 10 € per hour for study participation.

### Analyze of the fMRI behavioral data

Behavioral outcome of the fMRI task was estimated separately for the DD + imagination and the DD only conditions. The assumptions underlying the analyses of individual discount functions have been shown to largely influence the estimation of individual differences in discount rates. There is still no consensus regarding the mathematical form of the DD function in the literature, with main hypotheses describing hyperbolic or exponential discounting (Myerson and Green, 1995; Myerson et al., 2001; Rachlin, 2006; Takahashi et al., 2008; McKerchar et al., 2009; Peters et al., 2012). In this study, we applied an area-under-the-curve (AUC) index as the main behavioral outcome, since it is free of theoretical assumptions regarding the shape of the discounting, and is normally distributed (Myerson et al., 2001). The AUC value was calculated based on the empirically estimated six IP values for each condition. The normalized discount fraction (the fixed immediate reward value 20 € divided by IP) can be plotted against the normalized delay period (delay in days divided by 365 days). The AUC was calculated by summing up the areas of five trapezium shapes, from the plotted data, under the discount fractions. The AUC index varies between 0 and 1, where 0 indicates the highest (steepest) discount rate (preferring immediate choices) and 1 indicates minimal discount rate (preferring future choices). For the correlational imaging analyses, a difference score of the AUC index between the two conditions (DD + imagination vs. DD only) was calculated (indicated by ΔAUC). A larger difference score indicates a greater decrease in discount rate induced by the episodic future imagination, or a greater behavioral modulation effect.

Additionally, and to enable comparison with previous literature, we also calculated the discount factor according to a hyperbolic function (Green and Myerson, 2004; Kable and Glimcher, 2007, 2010; McKerchar et al., 2009; Peters et al., 2012). For estimating the hyperbolic discount factor, the behavioral data of each condition were modeled by a hyperbolic function (Mazur, 1987): value = A/(1 + *k*D), where “A” is the IP at each delay interval, “value” is the fixed immediate reward (20 €), “D” is the delay interval, and “*k*” is the discount parameter to be estimated. We used the curve fitting toolbox implemented in MATLAB (2013a, The MathWorks, Inc., Natick, MA USA), which uses a non-linear least square algorithm (*lsqcurvefit*), for the estimation of the “*k”* parameter. The discount rate was indicated by the log-transformed parameter ln(*k*). A higher ln(*k*) value indicates a greater (steeper) discount rate (preferring immediate choices). The hyperbolic function modeled k-parameter has been frequently used for modeling neural activity associated with subjective value (SV) of the future reward (Kable and Glimcher, 2007, 2010; Peters and Büchel, 2009). In the previous study, the condition specific hyperbolic k-parameter was used for generating the SV of the future reward, and the valuation signal was defined by the BOLD responses to the SV. These valuation signal changes were found to be associated with changes of discount rate between future imagination and control conditions (Peters and Büchel, 2010). To compare our data with this previous paradigm, we additionally applied a hyperbolic function for modeling neural responses related to SV (see Section The First-Level Analyses).

Finally, in order to verify if subjects have indeed chosen the future choices in about half of the trials as was planned by individually tailored future rewards, we calculated the percentage of future choices for each condition, where the higher percentage of future choices indicates a lower discount rate (preferring future choices).

### Image acquisition and preprocessing

Image acquisitions were performed using a 3 Tesla MRI scanner (Siemens Trio, Erlangen, Germany) with a standard 8-channel head coil. During the experiment an echo planar imaging (EPI) gradient echo sequence was used: voxel size = 3 × 3 × 3.75 mm^3^, 36 slices transversal acquisition, repetition time (TR) = 2.5 s, echo time (TE) = 30 ms, flip angle (FA) = 90°, field of view (FoV) = 192 × 192 × 135 mm^3^, matrix = 64 × 64. Field maps were acquired to correct any geometric distortions in EPI caused by static-field inhomogeneity using a gradient echo sequence with two echoes: voxel size = 3 × 3 × 3.75 mm^3^, 36 slices transversal acquisition, *TR* = 400 ms, *TE*(1) = 5.19 ms, *TE*(2) = 7.65 ms, *FA* = 60°, FoV = 192 × 192 × 135 mm^3^. A T1-weighted MP-RAGE sequence was used for structural MRI scans: *TR* = 1570 ms, *TE* = 3.42 ms, *TI* = 800 ms, *FA* = 15°, FoV = 256 × 256 × 160 mm^3^, matrix = 256 × 256, 160 slices, voxel size = 1 × 1 × 1 mm^3^.

Image analysis was preprocessed using SPM8 (http://www.fil.ion.ucl.ac.uk/spm/) implemented in MATLAB. The preprocessing of the EPI images included following steps: the origin set to the anterior commissure, generate the voxel displacement map by the field map toolbox, realignment and correction for field map distortion, slice timing, normalization to Montreal Neurological Institute (MNI) space using the “diffeomorphic anatomical registration through exponentiated lie algebra” (DARTEL) toolbox (Ashburner, 2007) and smoothing using a 10 mm full-width at half maximum (FWHM) Gaussian filter.

### fMRI statistics

#### The first-level analyses

FMRI statistics were calculated using the general linear model (GLM) as implemented in SPM8. We separately modeled the choice options into an initial period (0–3 s) and a later period (3–8 s) under either DD only or DD + imagination condition. We defined the 3 s as initial duration in order to be able to compare our results to the previous reported paradigm of Peters and Büchel (2010), that used a 3 s duration for choice option.

The first-level GLM included each period under each condition (i.e., DD only initial period, DD only later period, DD + imagination initial period, DD + imagination later period), the parametric modulations of the subjective value (SV) for each choice period under each condition as a regressor of interest, and the button press period as a regressor of non-interest. The parametric modulation of SV aims to model the brain activity that is related to the subjective value of the delayed reward (Kable and Glimcher, 2007; Peters and Büchel, 2009). Within the model, we included six covariates to capture residual movement related artifacts. Additionally, we included the time course of the average signal from white matter for each participant as an additional nuisance covariate in the design matrix to reduce global noise (Martin et al., 2015). For calculating the time series of the mean white matter signals, we thresholded individual white matter masks produced by the segmentation of T1 scan with a probability value of 0.99. Using the Marsbar toolbox (Brett et al., 2002), we read out the mean signal time course within the white matter volume from the realigned functional images. For each initial choice period regressor, sustained activation associated with the initial period (i.e., onsets of presentation of the choice options and lasting for 3 s) were modeled by boxcar regressors and convolved with the canonical hemodynamic response function. For each later choice period regressor, sustained activation associated with the later period (i.e., 3 s later than the onsets of presentation of the choice options and lasting for 5 s) were modeled by boxcar regressors and convolved with the canonical hemodynamic response function. The SV of each trial was calculated by multiplying the amount of the future reward with the empirically derived discount fraction (using hyperbolic *k*-parameters separated for each condition).

Altogether 12 contrast images were generated in the first-level analyses. Six contrasts coded for the choices periods: (1) DD only initial period; (2) DD only later period; (3) DD + imagination initial period; (4) DD + imagination later period; (5) imagination signal initial period (DD + imagination initial period vs. DD only initial period); (6) imagination signal later period (DD + imagination later period vs. DD only later period). Six contrasts coded for neural activity in response to SV (parametric modulation): (7) SV signal of DD only initial period; (8) SV signal of DD only later period; (9) SV signal of DD + imagination initial period; (10) SV signal of DD + imagination later period; (11) SV signal change initial period (SV signal of DD + imagination initial period vs. SV signal of DD only initial period); (12) SV signal change later period (SV signal of DD + imagination later period vs. SV signal of DD only later period).

#### The second-level analyses

The first-level contrasts (1–4), coding for the initial and later periods of intertemporal choices under both conditions, were entered into a second-level random effects analysis using correction for non-sphericity in the context of a flexible factorial design, for which a subject factor and a factor of condition type (i.e., DD only initial period, DD only later period, DD + imagination initial period, and DD + imagination later period) were specified. The neural activity associated with the initial period and the later period of the episodic future imagination was calculated by the categorical comparisons (DD + imagination initial period vs. DD only initial period, or DD + imagination later period vs. DD only later period). The brain activity associated with both periods of episodic future imagination was calculated by the conjunction of previous categorical comparisons. The neural activity uniquely associated with the initial period was calculated by interaction analysis [(DD + imagination initial period vs. DD only initial period) vs. (DD + imagination later period vs. DD only later period)]. And the brain activity uniquely associated with the later period was calculated by another interaction analysis [(DD + imagination later period vs. DD only later period) vs. (DD + imagination initial period vs. DD only initial period)].

To analyze the changes in the valuation process underlying the initial and later periods of future event imagination, the first-level contrasts (7–10), coding for the neural responses to SV under both periods under both conditions, were entered into a second-level random effects analysis using correction for non-sphericity in the context of a flexible factorial design, for which a subject factor and a factor of condition type (i.e., SV signal of DD only initial period, SV signal of DD only later period, SV signal of DD + imagination initial period, and SV signal of DD + imagination later period) were specified. Similar to the last flexible factorial design model using the first-level contrasts (1–4), categorical and conjunction analyses were also carried out for this design.

To analyze the correlation between changes of brain activation induced by episodic future imagination and the behavioral modulation effect, four different GLM models were calculated, by entering each of the first-level contrasts (5, 6, 11, or 12) as a dependent variable in each model, and with the behavioral modulation effect (ΔAUC) as an explanatory variable. We also calculated the GLM models using the behavioral modulation effect indicated by the other two indices [ln(*k*), percentage of future choices], which have revealed consistent results. Since the AUC value was considered the main outcome of this study, we present only the correlation results with the ΔAUC.

#### Psychophysiological interaction analysis

In the current study, the context dependent (DD + imagination vs. DD only) functional connectivity (FC) between a seed region and the rest of the brain areas was computed by using the generalized psychophysiological interactions (gPPI) toolbox (McLaren et al., 2012) (http://www.nitrc.org/projects/gppi). Seed regions were defined as the brain areas revealing significant correlations between the behavioral modulation effect and brain activity changes induced by future imagination. More specifically, the seed regions were individually defined as the sphere with 3 mm radius around the peak voxel of the first-level contrasts (5, 6, 11, 12), that is sphere with 10 mm radius of the peak activation of the corresponding second-level correlational analysis (see Section The Second-Level Analyses).

Using the gPPI toolbox, a design matrix of three sets of columns per run was created: (1) task regressors were formed by convolving the task blocks with the canonical hemodynamic response function; (2) BOLD signal observed from the seed region; and (3) PPI regressors that are formed by multiplying the tasks by the deconvolved BOLD signal derived from seed region, and then convolved with the canonical hemodynamic response function, separately for each task condition (either DD only or DD + imagination). In the present study, 5 regressors were included in the gPPI analysis, per run: (1) the task regressor of DD without future imagination (DD only); (2) the task regressor of DD with future imagination (DD + imagination); (3) BOLD signal in the PPI seed region; (4) PPI regressor under the DD only condition; (5) PPI regressor under the DD + imagination condition. The model also included the motion parameters and a regressor for the white matter signal (see Section The First-Level Analyses). Following the creation of the design matrix, the gPPI toolbox estimated the model parameters and computed linear contrasts. In this study, the linear contrast of PPI regressor between two conditions (DD + imagination vs. DD only) is calculated and used in the further second-level correlational analyses.

Correlational analyses were carried out between the PPI regressors (DD + imagination vs. DD only) and the behavioral modulation effect, as well as between the PPI regressors (DD + imagination vs. DD only) and the scores on the episodic memory capacity (the VLMT first learning trial, or the VLMT sum of five learning trials). These correlational analyses aim to test whether the context dependent FC between our seed regions and other brain areas covary with these behavioral measures (behavioral modulation effect, episodic memory capacity).

#### Statistical threshold

Generally, a family-wise error (FWE) corrected statistical threshold of *p* < 0.05 at the cluster level was adopted (height threshold: *T* = 3.29, *p* = 0.001; extent threshold: *k* = 350). Furthermore, region of interest (ROI) analyses over bilateral anterior cingulate cortex (ACC) and bilateral hippocampus were performed for the analyses regarding the subjective valuation analyses, since a hippocampal-ACC interaction was previously found to mediate the modulation of episodic future imagination on DD (Peters and Büchel, 2010). For these ROI analyses, a statistic threshold of *p* < 0.05 (FWE, small volume correction) using the anatomical masks of bilateral ACC and bilateral hippocampus was applied. The anatomical masks were generated by the WFU_Pickatlas toolbox (http://www.nitrc.org/projects/wfu_pickatlas/). An additional ROI analysis over ventral striatum was also performed for the analyses regarding the subjective value, since it has been shown to be a key region for subjective valuation in DD in several studies (Kable and Glimcher, 2007; Peters and Büchel, 2009, 2010). For this analysis, a statistic threshold of *p* < 0.05 (FWE, small volume correction) using the ROI of sphere with 10 mm radius around the MNI coordinate [10 8 0] (Peters and Büchel, 2010) was applied. For the exploratory purpose, the results of the gPPI analyses within the hippocampal areas were also reported if the significant level reached *p* < 0.005 (uncorrected).

### Hierarchical multiple regression analysis

Our fMRI statistics aim to test whether the behavioral modulation effect is associated with the changes of brain activations induced by episodic future imagination, as well as their connectivity with other brain areas. Since the hippocampus has been suggested to support decision making through interaction with cortical areas via multiple pathways (Buckner, 2010), those cortical areas, that are both interacting with the hippocampus, and correlate with the behavioral modulation effect, may be important cortical nodes for mediating the effect of episodic future imagination on intertemporal decisions. Currently, it is not clear whether these nodes have a unique or shared contribution to the behavioral modulation effect. Therefore, to test this effect we conducted a hierarchical multiple regression analysis within SPSS, specifying the behavioral modulation effect (ΔAUC) as the dependent variable. The cortical signal changes induced by the future imagination and that functionally interaction with hippocampal areas were added as independent variables. The Marsbar toolbox used to extract the BOLD signals. All the independent variables were entered in a stepwise manner (criteria of inclusion, *p* < 0.05; criteria of exclusion, *p* > 0.10; for the significant change of the F statistics).

## Results

### Behavioral results

All subjects reported that it was easy to imagine their future events in both the pre-scan and post-scan interviews (difficulty: pre = 3.1 ± 1.9; post = 3.3 ± 1.9). The imagination evoked positive emotions (emotion: pre = 7.7 ± 1.2; post = 7.4 ± 1.3) and moderate arousal (arousal: pre = 4.1 ± 2.2; post = 3.3 ± 1.9). No differences were found for the difficulty of imagination [*t*_(21)_ = −0.85, *p* > 0.05] and the intensity in the positive emotion [*t*_(21)_ = 1.63, *p* > 0.05] between the pre-scan and the post-scan interview; whilst arousal ratings from the post-scan interview were significantly lower than the pre-scan interview [*t*_(21)_ = 5.00, *p* < 0.0001].

The behavioral DD pretest was a self-paced decision making task guided by an adjustment procedure. In the current study, the number of trials needed to terminate the adjustment ranged 74–192 trials (median = 106 trials), which is similar to the number of trials needed in the previous study (Richards et al., 1999) (range 74–148 trials, median = 103 trials). The reaction time (RT) of the choices during the pretest was 2.9 ± 1.0 s. IP values were estimated for the DD pretest by using a binary logistic regression. The classification accuracy of future and immediate responses of our subjects using the estimated IP values was 92.7 ± 6.4%.

The hit rates of the fMRI task were 99.7 ± 0.9% for the DD only condition and 99.5 ± 1.1% for the DD + imagination condition. No difference was found for the hit rates [*t*_(21)_ = 0.67, *p* > 0.05] between two conditions. The mean RT values for button press responses were obtained for the choice periods after the jitter periods. The RT for the DD only condition was 726 ± 21 ms, and was 772 ± 22 ms for the DD + imagination condition. Peters and Büchel (2010) previously showed that RT may vary as a function of future reward value (lower, similar, or higher than the reference option value). In the current study, repeated-measures ANOVA of RT as a function of condition (DD only, or DD + imagination) and a function of future reward value (lower, similar, or higher relative to IP) showed no significant main effect of condition (p = 0.23), future reward value (*p* = 0.14), or interaction effect for reward value (i.e., condition × future: *p* = 0.19.

IP values were estimated for the behavioral responses during the fMRI task, separately for each delay period under each condition by using the binary logistic regression. The accuracy of classification of the future and immediate responses using the estimated IP values were 93.6 ± 4.7% for the DD only condition and 91.1 ± 6.1% for the DD + imagination condition. No difference was found for the classification accuracy between two fMRI conditions [*t*_(21)_ = 1.7, *p* > 0.05], as well as between the classification accuracy of the pretest results and the fMRI behavioral results [*t*_(21)_ < 1.58, *p* > 0.05].

AUC values were calculated based on the IP values separately for each condition. They show a significant increase in the DD + imagination condition, indicating an increased preference for future choices [DD only: 0.50 ± 0.18; DD + imagination: 0.53 ± 0.18; *t*_(21)_ = −2.2, *p* < 0.05]. These effects hold true when (1) estimating a hyperbolic discount parameter, showing significant steeper discounting in the DD only condition [DD only: −4.7 ± 1.2; DD + imagination: −4.9 ± 1.1; *t*_(21)_ = 2.3, *p* < 0.05], or (2) just looking at the percentage of choosing the future options [DD only: 43.5 ± 16.8%; DD + imagination: 49.0 ± 18.2%, *t*_(21)_ = −2.1, *p* < 0.05, Figure 1B].

### fMRI results: categorical, conjunction, and interaction analyses

**Figure 2:**
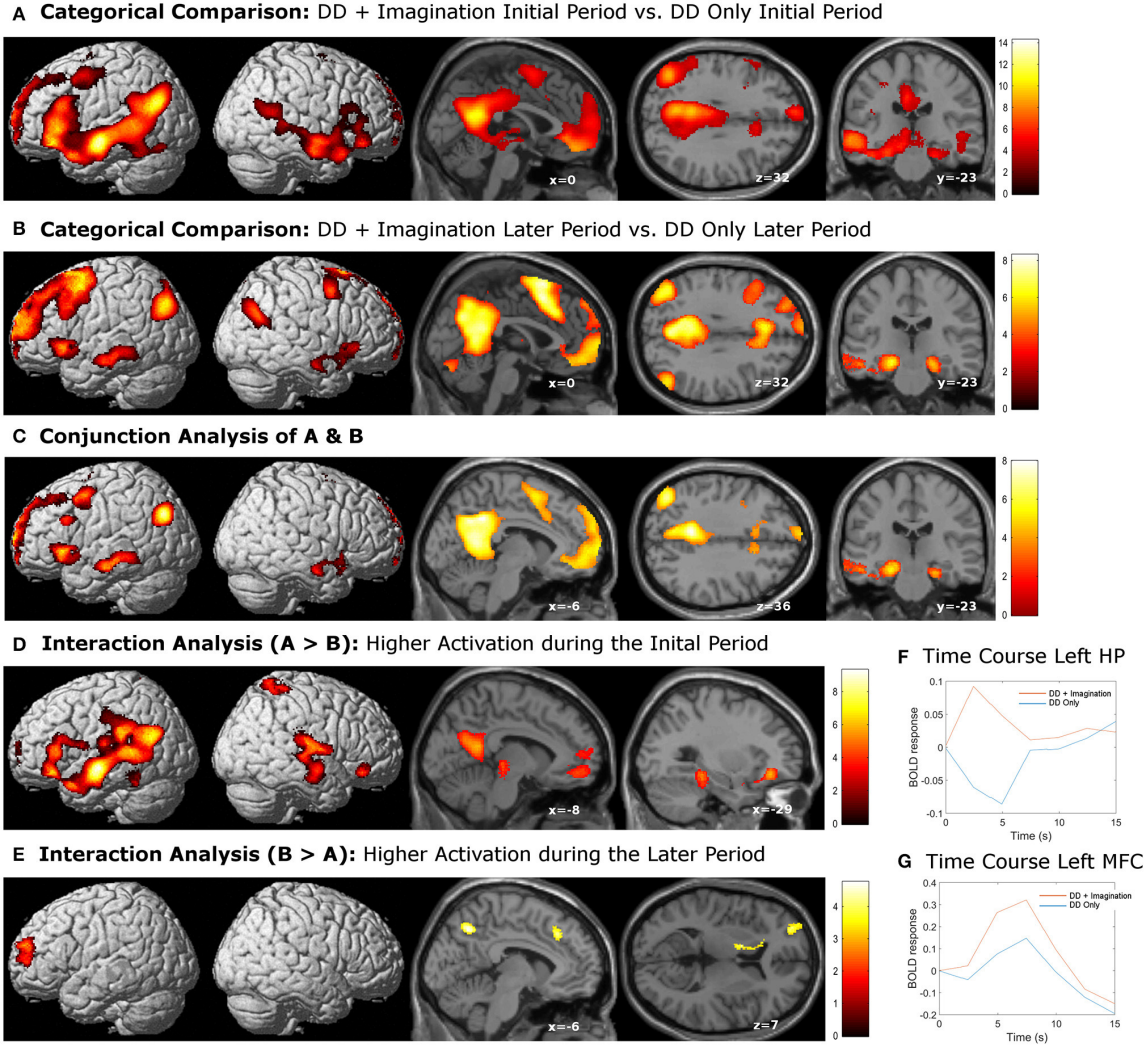
**Activations related to episodic future imagination. (A,B)** Categorical comparisons (DD + imagination vs. DD only) of the initial period (0–3 s) and the later period (3–8 s); **(C)** the conjunction analysis of **(A,B)**, which revealed a network akin to the default mode network; **(D)** interaction analysis that showed more activations in the bilateral temporal, frontal, and parietal corticies, precuneus extending to the left medial temporal lobe, and the left medial prefrontal cortex during the initial period than the later period; **(E)** interaction analysis that showed more activations in the bilateral percuneus, bilateral medial prefrontal cortices, left basal ganglia and left prefrontal cortex during the later period than the initial period. All results were sigificant at *p* < 0.05, whole brain cluster level family wise error (FWE) corrected. **(F)** mean time course of the peak voxel of left hippocampus (HP), which resulted from the interaction analysis **(D)**. **(G)** mean time course of the peak voxel of left middle frontal cortex (MFC), which resulted from the interaction analysis **(E)**.

The categorical comparison of the initial period of intertemporal choices between the two conditions (DD + imagination initial period vs. DD only initial period) showed neural activity in a network of prefrontal, temporal, inferior parietal, medial frontal, medial parietal, and medial temporal areas bilaterally (Figure 2A). The categorical comparison of the later period between two conditions (DD + imagination later period vs. DD only later period) revealed brain activity in a network of similar brain areas (Figure 2B). The conjunction analysis of both categorical comparisons revealed a network including retrosplenial cortex, PCC, ACC/mPFC, MTL (including hippocampus), temporal cortices, bilaterally, and left prefrontal and inferior parietal cortices (Figure 2C; Table 1). Interaction analysis showed more brain activity in response to the initial period in bilateral temporal cortices, bilateral inferior frontal cortices, left inferior parietal cortex (IPC), right superior parietal gyrus, lower part of the left precuneus extending to left MTL (including hippocampus), plus the lower part of left mPFC (Figure 2D; Table 2). Interaction analysis also revealed more activation during the later period in the upper part of the bilateral precuneus, upper part of the bilateral mPFC, left basal ganglia, and left prefrontal cortex (Figure 2E; Table 3). The fore mentioned results were significant at a threshold of p_FWE_ < 0.05 (whole brain cluster level corrected). To demonstrate the differential underlying processes associated with the initial period or the later period of the future imagination, we further conducted a time course analyses using the finite impulse response (FIR) model. For the time course analysis, we concentrated on two regions resulting from the aforementioned interaction analyses—the left hippocampus (Figure 2D) and the left middle frontal cortex (Figure 2E). The left hippocampus was chosen, for demonstrating the time course of the activity, since this was previously shown to be more activated during the construction than the elaboration period (Addis et al., 2007), and considered as the key region for event construction (Addis et al., 2011; Gaesser et al., 2013). The mean time course of the peak voxel of left hippocampus showed a peak at 2.5 s under the DD + imagination condition and dropped rapidly after the 2.5 s (Figure 2F). The left middle frontal cortex was chosen, to demonstrate the time course of the activity, because previous studies showed that lateral cortical regions are more involved in the elaboration period (Addis et al., 2007; Berryhill et al., 2010). The mean time course of the left middle frontal cortex showed rising signal after the 2.5 s and peaked at 7.5 s under the DD + imagination condition (Figure 2G).

**Table 1:** **Loci of BOLD responses associated with episodic future imagination in both initial period and later period – conjunction analysis [(DD + imagination initial period vs. DD only initial period) and (DD + imagination later period vs. DD only later period)] (Height threshold: *T* = 3.29, *p* = 0.001; extent threshold: *k* = 350)**.

**Locations**	**Cluster size**	**Peak MNI coordinates**			***T***	***Z***
		***x***	***y***	***z***		
Left middle cingulate cortex	11382	−6	−46	36	7.95	6.85
Right precuneus		8	−48	19	7.52	6.56
Right calcarine		6	−54	12	7.46	6.52
Left precuneus		−6	−61	33	7.40	6.47
Left posterior cingulate cortex		−2	−48	24	7.11	6.27
Left middle occipital cortex	1900	−42	−72	34	6.87	6.10
Left inferior parietal cortex		−45	−70	33	6.95	5.90
Right medial orbitofrontal cortex	12239	8	57	−12	6.56	5.88
Left superior medial frontal cortex		−3	66	4	5.96	5.42
Left medial orbitofrontal cortex		−3	60	−2	5.66	5.20
Left middle frontal cortex		−20	24	45	5.49	5.06
Left superior frontal cortex		−17	24	42	5.08	4.73
Left middle cingulate cortex		−11	17	40	4.97	4.64
Left supplementary motor area		−9	9	52	4.95	4.63
Right middle cingulate cortex		9	20	31	4.81	4.50
Right supplementary motor area		3	2	62	5.26	4.88
Left parahippocampus	3424	−23	−28	−17	6.25	5.65
Left hippocampus		−20	−27	−11	5.57	5.13
Left middle temporal gyrus		−54	−18	−17	4.99	4.66
Left fusiform gyrus		−33	−28	−20	3.86	3.70
Left superior temporal pole	2273	−53	14	−12	5.38	4.97
Left insula		−27	23	3	5.37	4.96
Left inferior frontal cortex		−54	21	−8	5.26	4.88
Left putamen		−20	14	3	4.11	3.91
Right parahippocampus	863	23	−19	−20	5.13	4.77
Right fusiform gyrus		24	−30	−18	5.09	4.73
Right parahippocampus		20	−34	−11	3.91	3.73
Right hippocampus		24	−14	−23	3.51	3.38
Right middle temporal gyrus	610	56	−12	−21	4.28	4.06
Right middle temporal pole		57	14	−9	3.85	3.68
Right superior temporal gyrus		63	−1	−12	3.70	3.55
Left inferior frontal cortex	597	−45	20	28	4.11	3.91
Left middle frontal cortex		−32	8	33	3.35	3.24

*MNI, Montreal-Neurological-Institute*.

**Table 2:** **Loci of BOLD responses more associated with initial period of episodic future events – interaction analysis [(DD + imagination initial period vs. DD only initial period) vs. (DD + imagination later period vs. DD only later period)] (Height threshold: *T* = 3.29, *p* = 0.001; extent threshold: *k* = 350)**.

**Locations**	**Cluster size**	**Peak MNI coordinates**			***T***	***Z***
		***x***	***y***	***z***		
Left middle temporal gyrus	17963	−57	−9	−11	9.29	7.68
Left inferior frontal cortex		−38	32	−14	7.80	6.75
Left middle temporal pole		−47	15	−27	5.92	5.40
Left inferior parietal cortex		−51	−67	25	4.33	4.10
Left supramarginal gyrus		−60	−43	27	4.12	3.92
Left inferior temporal gyrus	5108	−39	−37	−15	8.60	7.26
Left precuneus		−8	−51	16	5.80	5.31
Left cuneus		−9	−64	24	5.36	4.96
Left parahippocampus		−29	−34	−12	5.41	5.00
Left fusiform gyrus		−29	−34	−18	4.84	4.53
Left hippocampus		−30	−33	−12	4.75	4.46
Right inferior frontal cortex	750	36	35	−12	7.80	6.75
Right rolandic operculum	5251	49	−10	10	5.47	5.04
Right superior temporal gyrus		59	−6	−9	4.73	4.44
Right middle temporal gyrus		53	−12	−14	4.67	4.39
Right inferior temporal gyrus		54	−16	−23	4.36	4.13
Right superior parietal gyrus	1017	26	−55	70	4.67	4.39
Left medial superior frontal cortex	1318	−12	63	12	4.50	4.24
Left medial orbitofrontal cortex		−6	50	−14	4.40	4.16
Left medial superior frontal cortex		−11	53	7	4.16	3.96

*MNI, Montreal-Neurological-Institute*.

**Table 3:** **Loci of BOLD responses more associated with later period of episodic future imagination – interaction analysis [(DD + imagination later period vs. DD only later period) vs. (DD + imagination initial period vs. DD only initial period)] (Height threshold: *T* = 3.29, *p* = 0.001; extent threshold: *k* = 350)**.

**Locations**	**Cluster size**	**Peak MNI coordinates**			***T***	***Z***
		***x***	***y***	***z***		
Left precuneus	579	−6	−60	45	4.75	4.46
Right precuneus		5	−63	45	4.51	4.25
Left superior medial frontal cortex	480	−5	27	40	4.40	4.16
Right superior medial frontal cortex		2	26	46	3.61	3.47
Right middle cingulate cortex		6	32	33	3.55	3.42
Left pallidum	669	−14	5	7	4.23	4.02
Left caudate		−14	24	3	4.22	4.01
Left middle frontal cortex	1025	−29	51	22	4.14	3.94
Left superior frontal cortex		−26	59	25	3.68	3.53

*MNI, Montreal-Neurological-Institute*.

**Figure 3:**
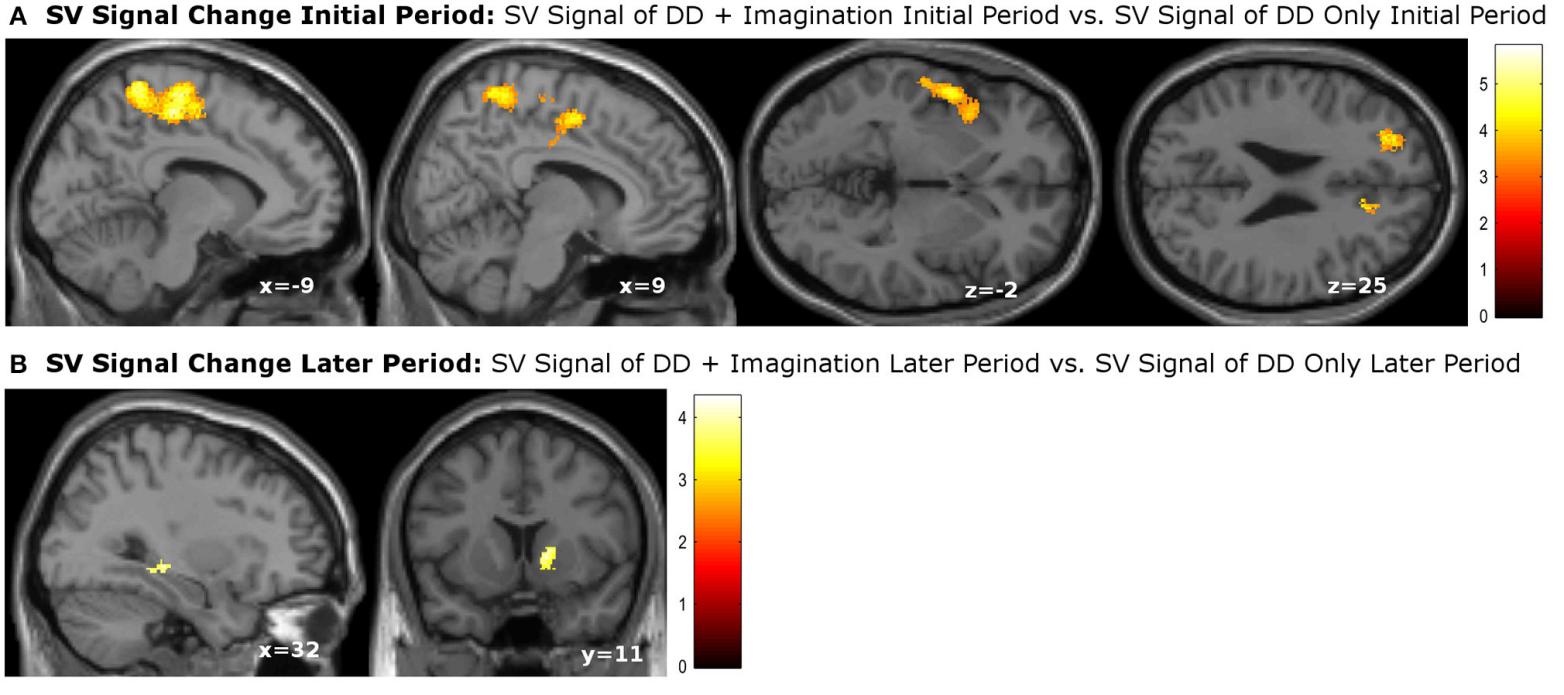
**subjective value (SV) signal changes due to future imagination. (A)** SV signal changes in the bilateral precuneus, bilateral middle cingulate cortex, left insula cortex, left superior temporal cortex, left prefrontal cortex, and right anterior cingualte cortex (ACC) during the initial period; **(B)** SV change in the right hippocampus, and right ventral striatum during the later epriod. The activations in the right ACC, right hippocampus were corrected at *p* < 0.05, family wise error (FWE) small volume corrected using the anatomical ROI of bilateral ACC or bilateral hippocampus. The activation in the right ventral striatum was signfiicant at *p* < 0.05, FWE small volume corrected using sphere with 10 mm radious centered at MNI coordinate [10 8 0]. Other activations were significant at *p* < 0.05, whole brain cluster level FWE corrected.

We then performed categorical analysis of the SV signals under each condition. SV signals resulted from the parametric modulation of SV, referring to the association between the BOLD response in a specific brain area and the subjective value of future reward. The “SV signal change” refers to the change of such associations between two conditions in a specific brain area (or this area has different responsiveness to SV between two conditions). Previous studies show that different regions may respond differently to subjective values under various conditions. For example, different valuation areas may be specifically responsive to positive subjective value or negative subjective value (Bartra et al., 2013), whether the decision process involves the integration of value and costs (Massar et al., 2015), or the reward modality (e.g., food vs. money) (Clithero and Rangel, 2014). Previous studies using the P&B paradigm (Peters and Büchel, 2010) found BOLD responses to SV in various brain areas including the ACC, the left parietal cortex, and the amygdala. Only the change of SV signals in ACC significantly varied with the change of behavioral discount rate. To enable comparison of our data with previous research, we performed the SV analysis in the same manner as in the P&B paradigm. The SV signal change in the initial period (SV signal of DD + imagination initial period vs. SV signal of DD only initial period) were found in bilateral precuneus, bilateral middle cingulate cortices, left insula cortex and its adjacent superior temporal gyrus, left prefrontal cortex (p_FWE_ < 0.05, whole brain corrected), as well as a cluster in right ACC (p_FWE_ = 0.004, small volume corrected, using the anatomical ROI of the bilateral ACC) (Figure 3A; Table 4). The SV signal change in the later period (SV signal of DD + imagination later period vs. SV signal of DD only later period) were found in the right hippocampus (p_FWE_ = 0.031, small volume corrected, using the anatomical ROI of bilateral hippocampus), and in right ventral striatum (p_FWE_ = 0.003, small volume corrected using the functional ROI, sphere with 10 mm radius centered at the MNI coordinate [10 8 0]) (Figure 3B; Table 5). The conjunction analysis between the two fore mentioned analyses showed no significant voxel.

**Table 4:** **Loci of subjective value signal changes during the initial period (Height threshold: *T* = 3.29, *p* = 0.001; extent threshold: *k* = 350)**.

**Locations**	**Cluster size**	**Peak MNI coordinates**			***T***	***Z***
		***x***	***y***	***z***		
Left precuneus	6922	−9	−45	66	5.17	4.80
Right precuneus		9	−45	63	4.65	4.37
Right middle cingulate cortex		2	−12	35	4.67	4.39
Left middle cingulate cortex		−2	0	35	4.25	4.04
Left superior temporal gyrus	771	−53	3	−6	4.99	4.65
Left insula cortex		−39	12	−2	4.15	3.95
Left middle frontal cortex	436	−24	47	24	4.89	4.57
Left superior frontal cortex		−20	50	16	3.73	3.58
Right anterior cingulate cortex^*^	89	15	33	25	4.79	4.49

*MNI, Montreal-Neurological-Institute*.

* *This area encompassed the small volume correction using the anatomical region of interest of bilateral anterior cingulate cortex, p_FWE_ = 0.004*.

**Table 5:** **Loci of subjective value signal change during the later period (Height threshold: *T* = 3.29, *p* = 0.001)**.

**Locations**	**Cluster size**	**Peak MNI coordinates**			***T***	***Z***
		***x***	***y***	***z***		
Right ventral striatum^*^	241	15	11	1	4.33	4.10
Right hippocampus^#^	77	30	−33	−6	4.00	3.82

*MNI, Montreal-Neurological-Institute*.

* *This area encompassed the small volume correction using region of interest of right ventral striatum, either using the 10 mm sphere around the coordinate [10 8 0]) based on the result of Peters and Büchel (2010), p_FWE_ = 0.003, or using the 10 mm sphere around the coordinate [11 5 1] based on the result of Kable and Glimcher (2007), p_FWE_ = 0.003*.

# *This area encompassed the small volume correction using the anatomical region of interest of bilateral hippocampus, p_FWE_ = 0.031*.

### fMRI results: correlations of the behavioral modulation effect and brain signal changes

**Figure 4:**
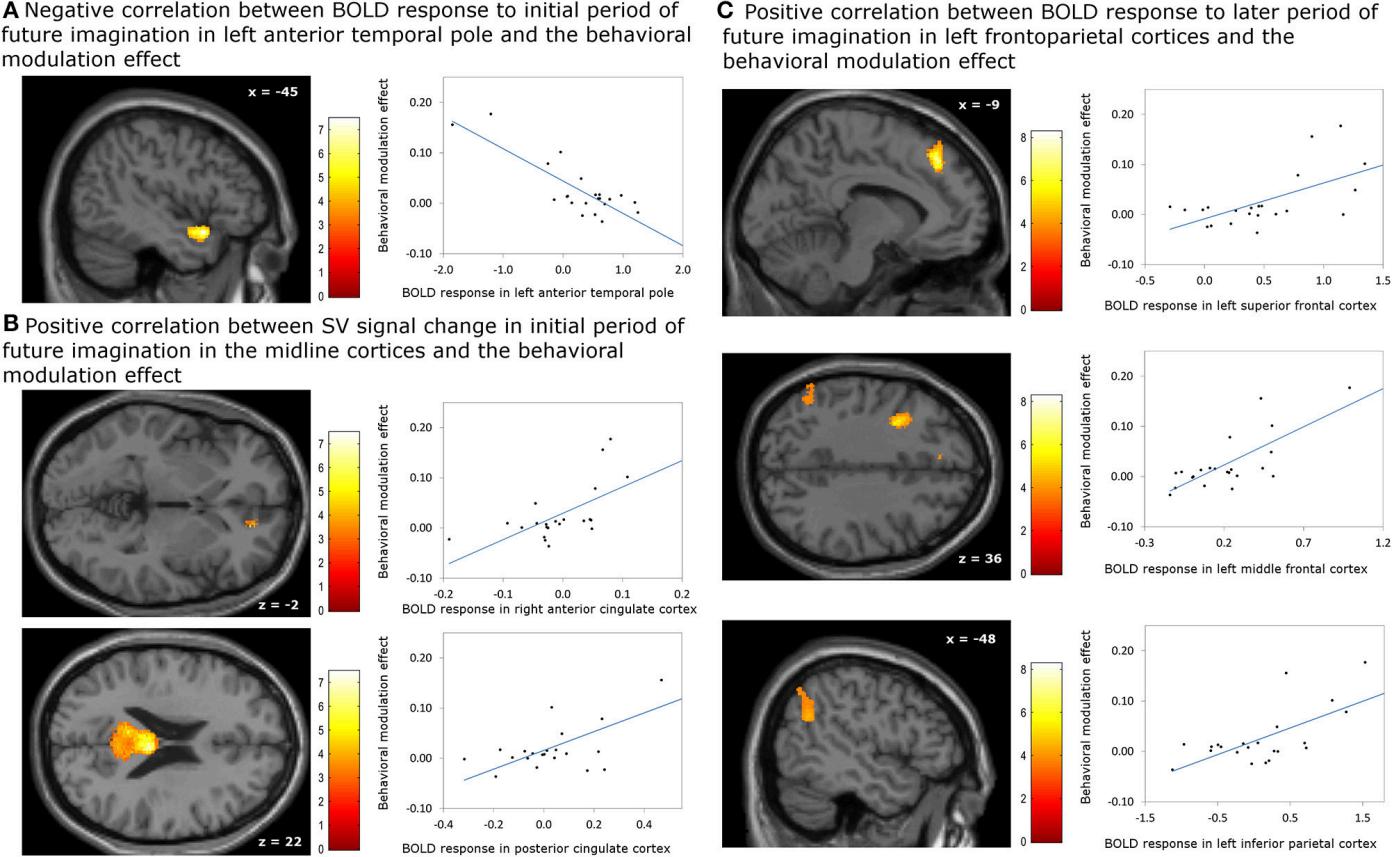
**Correlation between BOLD responses due to episodic future imagiantion and behavioral modulation effect. (A)** Negative association with the BOLD response to initial period (0–3 s) in left anterior temporal pole; **(B)** positive association with the subjective value (SV) signal change during the initial period (0–3 s) in the right anterior cingulate cortex (ACC) and bilateral posterior cingulate cortex; **(C)** positive assocation with the BOLD responses to later period (3–8 s) in left frontoparietal corticies. The result in the right ACC was significant at *p* < 0.05, family wise error (FWE) small volume corrected using the anatomical ROI of bilateral ACC. Other results were significant at *p* < 0.05, whole brain cluster level FWE corrected.

The analyses regarding the correlations between the behavioral modulation effect (ΔAUC) and the neuronal signal changes induced by the episodic future imagination revealed the following results. First, a negative association was found between the behavioral modulation effect and the brain activity associated with the initial period of future imagination in the left anterior temporal pole (Figure 4A). Second, during the initial period, positive correlations were found between the behavioral modulation effect and SV signal change due to imagination in the right ACC and in the bilateral PCC (Figure 4B). Third, positive correlations were found between the behavioral modulation effect and the neural activity associated with the later period of future imagination in the left superior and middle frontal cortices, and the left IPC (Figure 4C). The aforementioned results were significant at the threshold of p_FWE_ < 0.05 (whole brain corrected), except for the association in the ACC (p_FWE_ < 0.05, small volume corrected, using anatomical ROI of bilateral ACC) (Table 6). All these areas were later used as the seed regions for the gPPI analyses.

**Table 6:** **Significant correlation between the behavioral modulation effect (Δ AUC) and the change of neural responses due to episodic future imagination (Height threshold: *T* = 3.29, *p* = 0.001; extent threshold: *k* = 350)**.

**Locations**	**Cluster size**	**Peak MNI coordinates**			***T***	***Z***
		***x***	***y***	***z***		
**NEGATIVE ASSOCIATION WITH NEURAL RESPONSE TO IMAGINATION DURING THE INITIAL PERIOD (0–3 s)**						
Left anterior temporal pole	714	−45	11	−17	7.45	5.10
**POSITIVE ASSOCIATION WITH SUBJECTIVE VALUE SIGNAL CHANGE DURING THE INITIAL PERIOD (0–3 s)**						
Corpus callosum	2458	6	−27	22	7.14	4.98
Right posterior cingulate cortex		1	−28	28	6.40	4.67
Left posterior cingulate cortex		−5	−34	24	5.64	4.31
Right anterior cingulate cortex^*^	69	17	45	−2	5.32	4.15
**POSITIVE ASSOCIATION WITH NEURAL RESPONSE TO IMAGINATION DURING THE LATER PERIOD (3–8 s)**						
Left superior frontal cortex	870	−9	36	43	8.25	5.38
		−2	39	51	5.36	4.17
		−20	23	52	4.39	3.63
Left middle frontal cortex	489	−33	8	36	5.69	4.34
		−35	15	39	4.14	3.48
Left inferior parietal cortex	447	−48	−57	28	4.58	3.75
		−59	−55	39	4.25	3.56
		−53	−60	46	4.03	3.40

MNI, Montreal-Neurological-Institute; Δ AUC, increases in the area under the curve;

* *These areas encompassed the small volume correction using anatomical region of interest of the bilateral anterior cingulate cortex, p_FWE_ = 0.020*.

### fMRI results: gPPI analysis

**Figure 5:**
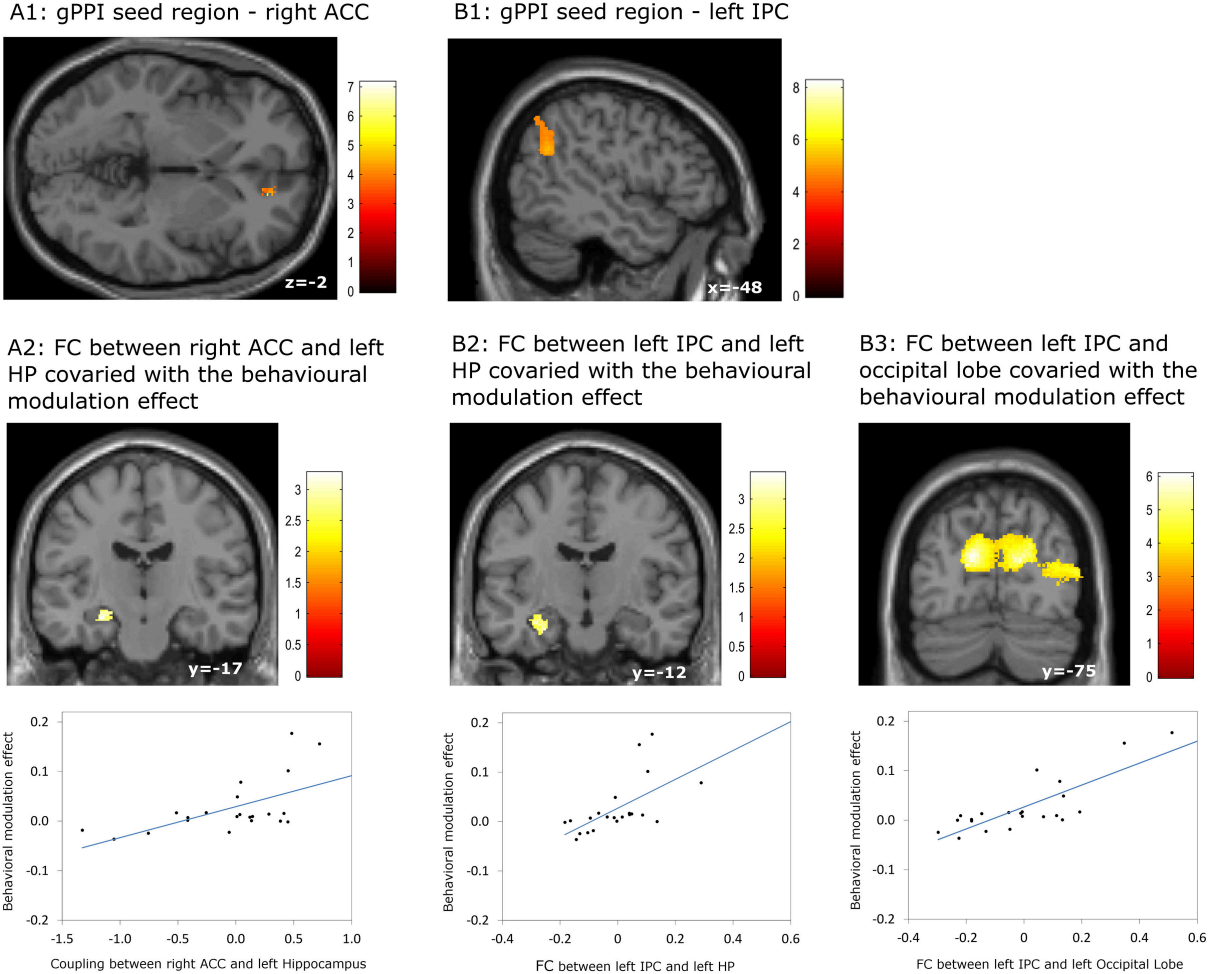
**Results of the generalized psychophysiological interaction (gPPI) analyses which revealed associations between the behavioral modulation effect and functional connectivity (FC) orginiating from the right anterior cingulate cortex (ACC) and the left inferior parietal cortex (IPC)**. **(A1,A2)** FC between right ACC and left hippocampus (HP) covaried with the behavioral modulation effect; **(B1–B3)** FC bewteen left IPC and left HP, between left IPC and bilateral occipital lobe, covaried with the behavioral modulation effect. Results regarding left HP were signficant at *p* < 0.001, uncorrected, whereas other results were significant at *p* < 0.05, whole brain cluster level family wise error corrected.

Our gPPI analyses revealed positive correlations between the behavioral modulation effect and the context dependent (DD + imagination vs. DD only) connectivity between the right ACC (Figure 5A1) and the left hippocampus (*p* = 0.002, uncorrected) (Figure 5A2), between the left IPC (Figure 5B1) and the left hippocampus (*p* = 0.001, uncorrected) (Figure 5B2), and between the left IPC (Figure 5B1) and bilateral occipital lobe (p_*FWE*_ < 0.05, whole brain corrected) (Figure 5B3; Table 7). No significant correlations were found between the behavioral modulation effect and the FC between other seed regions (left anterior temporal pole, PCC, left superior frontal cortex, and left middle frontal cortex) and the rest of the brain areas.

**Table 7:** **Significant correlation between the behavioral modulation effect (Δ AUC) and the context dependent (DD + imagination vs. DD only) functional connectivity using generalized psychophysiological analysis (Height threshold: *T* = 2.85, *p* = 0.005)**.

**Locations**	**Cluster size**	**Peak MNI coordinates**			***T***	***Z***
		***x***	***y***	***z***		
**POSITIVE ASSOCIATION WITH THE FUNCTIONAL CONNECTIVITY OF RIGHT ACC**						
Left hippocampus	59	−24	−17	−15	3.26	2.89
**POSITIVE ASSOCIATION WITH THE FUNCTIONAL CONNECTIVITY OF LEFT IPC**						
Left hippocampus	65	−30	−12	−20	3.43	3.01
Left superior occipital gyrus^*^	4747	−15	−75	21	6.06	4.51
Left cuneus^*^		−14	−77	23	5.94	4.46
Right cuneus^*^		17	−74	20	5.88	4.43
right middle occipital gyrus^*^		33	−72	12	5.25	4.11

MNI, Montreal-Neurological-Institute; Δ AUC, increases in the area under the curve;

* *These areas belong to the same cluster at the height threshold of T = 3.29, p < 0.001, and survive the significance level of p < 0.05 after the whole brain cluster level family-wise error (FWE) correction*.

**Figure 6:**
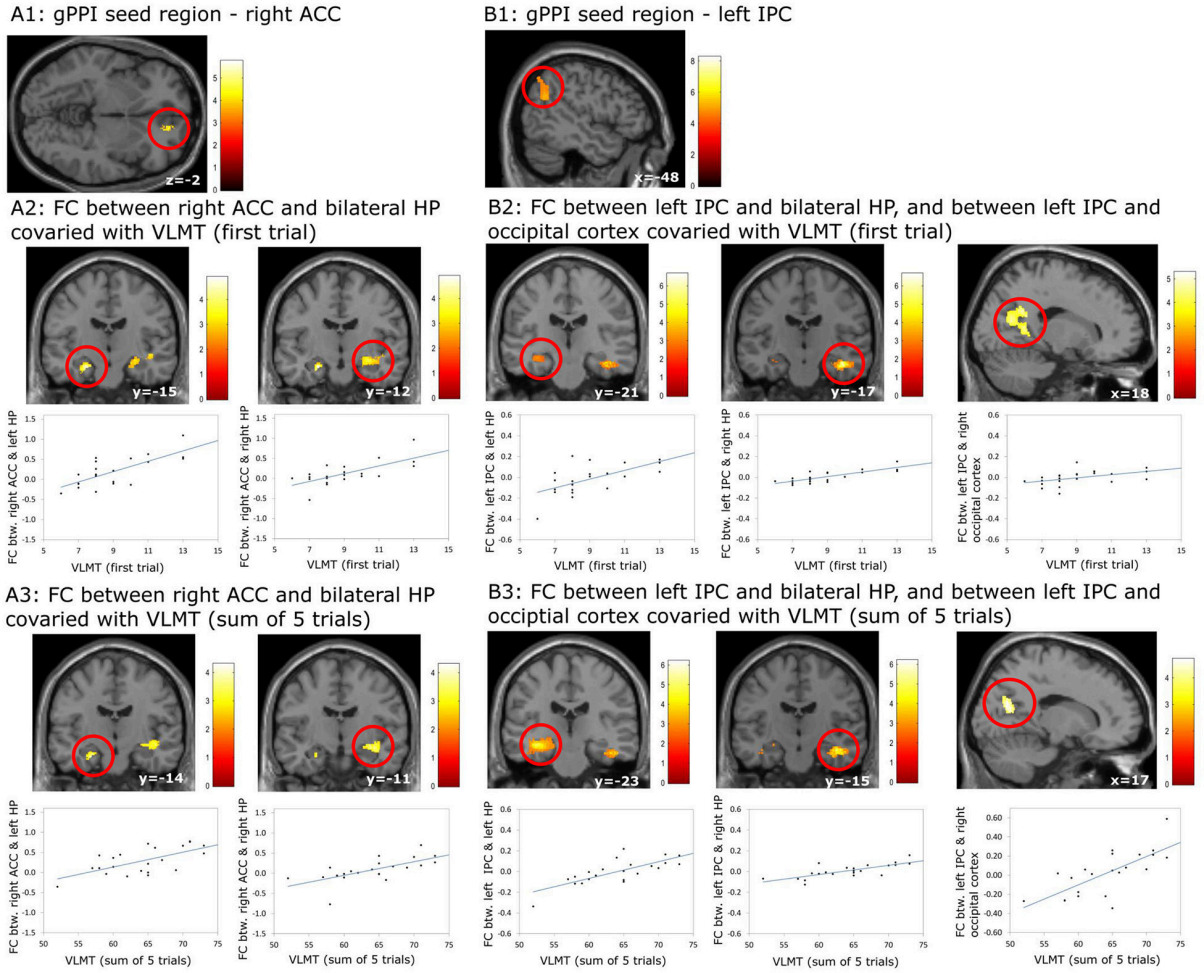
**Results of the generalized psychophysiological interaction (gPPI) analyses which revealed associations between the episodic memory capacity and functional coupling (FC) originating from the right anterior cingulate cortex (ACC) and the left inferior pareital cortex (IPC)**. **(A1–A3)** FC between right ACC and bilateral hippocampus (HP) covaried with the scores on episodic memory capacity; **(B1–B3)** FC between left IPC and bilateral HP, and between left IPC and occipital lobe covaried with the scores on episodic memory capacity. The results regarding the HP were significant at *p* < 0.001, uncorrected, whereas the results regarding the occipital lobe were significant at *p* < 0.05, whole brain cluster level family wise error corrected.

Using the gPPI analyses, the following positive associations between episodic memory capacity and FC originating from the right ACC (Figure 6A1) and the left IPC (Figure 6B1) were found (Table 8): (1) association between the VLMT score (first learning trial) and the FC between the right ACC and bilateral hippocampal regions (left: p_FWE_ < 0.036, small volume correlation, using the anatomical ROI of bilateral hippocampus; right: *p* < 0.001, uncorrected) (Figure 6A2); (2) association between the VLMT score (sum of 5 learning trials) and the FC between the right ACC and bilateral hippocampal regions (left: *p* = 0.001, uncorrected; right: *p* < 0.001, uncorrected) (Figure 6A3); (3) association between the VLMT score (first learning trial) and the FC between the left IPC and bilateral hippocampal regions (left: *p* = 0.001, uncorrected; right: p_*FWE*_ = 0.004, small volume correlation using the anatomical ROI of bilateral hippocampus), and the FC between the left IPC and the right occipital cortex (p_*FWE*_ < 0.001, whole brain corrected) (Figure 6B2); (4) associations between the VLMT score (sum of 5 learning trials) and the FC between the left IPC and bilateral hippocampus (left: p_FWE_ = 0.017; right: p_*FWE*_ = 0.018; small volume corrected, using the anatomical ROI of bilateral hippocampus), and the FC between the left IPC and the bilateral occipital cortices (p_*FWE*_ < 0.001, whole brain corrected) (Figure 6B3).

**Table 8:** **Significant correlation between the individual episodic memory capacity and the context dependent (DD + imagination vs. DD only) functional connectivity using generalized psychophysiological analysis (Height threshold: *T* = 2.85, *p* = 0.005)**.

**Locations**	**Cluster size**	**Peak MNI coordinates**			***T***	***Z***
		***x***	***y***	***z***		
**POSITIVE ASSOCIATION BETWEEN VLMT (FIRST TRIAL) AND FUNCTIONAL CONNECTIVITY OF RIGHT ACC**						
Left hippocampus^*^	159	−23	−15	−21	4.81	3.88
Right hippocampus	499	30	−12	−12	4.34	3.60
**POSITIVE ASSOCIATION BETWEEN VLMT (SUM OF 5 TRIALS) AND FUNCTIONAL CONNECTIVITY OF RIGHT ACC**						
Left hippocampus	49	−26	−14	−23	3.77	3.25
Right hippocampus	53	33	−11	−14	3.94	3.35
**POSITIVE ASSOCIATION BETWEEN VLMT (FIRST TRIAL) AND FUNCTIONAL CONNECTIVITY OF LEFT IPC**						
Left hippocampus	62	−32	−21	−15	3.44	3.02
Right hippocampus	93	41	−17	−20	5.96	4.47
Right precuneus^a^	6855	18	−53	24	4.50	3.69
Right calcarine^a^		18	−65	17	4.58	3.75
Right lingual^a^		15	−50	6	5.01	3.99
**POSITIVE ASSOCIATION BETWEEN VLMT (SUM OF 5 TRIALS) AND FUNCTIONAL CONNECTIVITY OF LEFT IPC**						
Left hippocampus^†^	513	−33	−23	−14	5.12	4.05
Right hippocampus^†^	92	41	−15	−20	5.10	4.04
Right cuneus^b^	850	17	−68	21	4.71	3.81
Right precuneus^b^		5	−75	41	4.55	3.73
Left cuneus^b^		−15	−84	33	4.19	3.51
Right superior occipital cortex^c^	330	24	−83	30	4.64	3.78
Right cuneus^c^		20	−78	44	3.76	3.23

*MNI, Montreal-Neurological-Institute; ACC, anterior cingulate cortex; IPC, inferior parietal cortex*.

* *This area encompassed the small volume correction using anatomical region of interest of the bilateral hippocampus, p_FWE_ = 0.036*.

a, b, c *These areas belong to the same cluster at the height threshold of T = 3.29, p < 0.001, and survive the significance level of p_FWE_ < 0.05, whole brain cluster level corrected*.

† *These areas encompassed the small volume correction using anatomical region of interest of the bilateral hippocampus, left hippocampus—p_FWE_ = 0.017, right hippocampus—p_FWE_ = 0.018*.

### Hierarchical multiple regression results

Our fMRI results revealed two important cortical nodes, the right ACC and the left IPC. The brain activity and their context dependent (DD + imagination vs. DD only) FC with the left hippocampus significantly correlated with the behavioral modulation effect. The first model of the hierarchical multiple regression analysis included the SV signal change in the initial imagination period in the right ACC as the independent variable. This model revealed that the SV signal change in the right ACC was a significant predictor of the behavioral modulation effect [Table 9, Model 1; *R*^2^ = 0.39, Δ*F*_(1, 20)_ = 12.89, *p* < 0.01]. The second model additionally included the brain activity of the left IPC associated with the later imagination period as an additional independent variable. This additional independent variable significantly changed the goodness-of-fit of the linear model [Table 9, Model 2; *R*^2^ = 0.61, Δ*R*^2^ = 0.22, Δ*F*_(2, 19)_ = 10.82, *p* < 0.01]. The second model revealed that both the SV signals in the right ACC during the initial period and the neural responses to later imagination period in the left IPC were significant predictors (right ACC: *t* = 2.97, ß = 0.45, *p* < 0.01; left IPC: *t* = 3.29, ß = 0.50, *p* < 0.01) of the behavioral DD change.

**Table 9:** **Hierarchical regression analyses evaluating the predictor of subjective value signal change in right ACC during the initial period (0–3 s) and the activation in left IPC response to the later imagination period (3–8 s) on the behavioral modulation effect (Δ AUC)**.

**Measures**	***R***	***R*^2^**	**Δ*R*^2^**	**Δ*F***	***df***	***t***	**ß**
Model 1	0.63	0.39	0.39	12.89^**^	1.20		
Subjective value signal change in right ACC during the initial period						3.59	0.63^**^
Model 2	0.78	0.61	0.22	10.82^**^	2.19		
Subjective value signal change in right ACC during the initial period						2.97	0.45^**^
Neural response in the left IPC during the later imagination period						3.29	0.50^**^

*ACC, anterior cingulate cortex; IPC, inferior parietal cortex*.

** *p < 0.01*.

## Discussion

The current study demonstrated decreases of discount rates in an intertemporal choice task due to episodic future imagination. This behavioral modulation effect is consistent with previous findings showing that future imagination made people less myopic (Ungemach et al., 2011; Cheng et al., 2012; Daniel et al., 2013; Lin and Epstein, 2014; Dassen et al., 2016). The observed ln(*k*) value in the current study was comparable to a logarithmically transformed hyperbolic discount parameter observed in previous studies (Lin and Epstein, 2014; Dassen et al., 2016). The mean reaction times in the DD fMRI task were not different between conditions or across the choice options with different future reward values (lower, similar, or higher relative to IP), which is different from the previous report with similar paradigm (Peters and Büchel, 2010). This difference may be due to the prolonged period of the intertemporal decision (with and without future imagination) in the current paradigm (8 s instead of 3 s). Indeed, the mean RT of the intertemporal choice in our self-paced DD pretest was 2.9 ± 1.0 s, close to the decision period of the previous paradigm (3 s), and much shorter than the decision period of the current paradigm (8 s), indicating that our subjects had sufficient time to thoroughly think about their choice options before entering their decisions. The prolongation of the decision and imagination period has a further advantage, providing an additional period to allow enriched elaboration of the imagined future event. The current study also promoted our subjects to elaborate on their imagined event by using a detailed pre-scan interview (modified from Levine et al., 2002), and an explicit instruction of using vivid mental imagery during the future imagination task in the scanner. These are two important modifications to the P&B paradigm. Resulting from this preparation, our subjects reported that it was easy for them to imagine the future events both during the detailed pre-scan interview and during the scanning session, with no difference in the perceived difficulty levels or the emotional experience. This indicates that the event construction process during the imagination in the fMRI experiment was not demanding for our subjects and that they devoted the same degree of effort for imagination in the fMRI session and in the pre-scan interview. The same level of emotional experience may be accompanied by similar level of imagined details between the pre-scan interview and the fMRI session, indicating that our subjects may have used the full imagination period (8 s) for elaborating on their events during the scanning sessions. The following discussions on the neuroimaging results will shed more light on the underlying mechanism of the beneficial effect of elaborative future imagination on the DD.

Our fMRI results showed positive associations between the behavioral modulation effect and activation changes in the regions associated with valuation processes (right ACC and PCC) during the initial period of imagination (0–3 s); in addition, we also observed positive correlations between the behavioral modulation effect and the activity changes in left prefrontoparietal areas during the later period of imagination (3–8 s). The gPPI analyses, using these areas as the seed regions, showed positive associations between the behavioral modulation effect and the FC between the right ACC and the left hippocampus, between the left IPC and the left hippocampus, and between the left IPC and the bilateral occipital lobe. Connectivity among these areas was also correlated with individual episodic memory capacity. Finally, the hierarchical multiple regression analysis showed that both the activation changes in the right ACC and in the left IPC together significantly predicted the behavioral modulation effect. Our imaging results replicate previous findings with similar paradigms (Peters and Büchel, 2010; Benoit et al., 2011). Using our design with a prolonged and more elaborated imagination period, we additionally demonstrate that multiple hippocampal-cortical pathways are involved in this modulation effect, and the strength of these pathways was associated with the episodic memory capacity. The current results have some important clinical implications. With regard to human diseases, increased discount rates have been observed across a broad range of psychiatric disorders (e.g., addiction, schizophrenia, mild cognitive impairment, etc.); whereas the underlying neuronal mechanism of the DD deficits may be different. Applying a prolonged and more elaborated future imagination as a behavioral modulation strategy, multiple hippocampal—cortical pathways are to be activated, providing more neuronal interfaces for counteracting the tendency of short-sighted decisions in various psychiatric diseases. Second, the strength of the hippocampal—cortical pathways was found to be associated with episodic memory capacity. Note that some patients with memory deficits showed also increased discount rates (Lebreton et al., 2013; Lindbergh et al., 2014). For these subjects with episodic memory deficits, the episodic future imagination might not be a proper strategy for the intervention of their myopic decision preference. Other behavioral modulation strategies such as working memory training may be an alternative way of intervention (Bickel et al., 2011). In the following, we discuss our imaging results in more detail.

The categorical comparisons of the choice process between the DD + imagination and DD only conditions revealed that similar brain networks are engaged in the initial and later periods of future imagination, but to a different extent. The conjunction analysis of both periods revealed a “core” network including the bilateral mPFC, retrosplenial cortex, posterior parietal, middle and medial temporal cortices (including the bilateral hippocampus), and the left prefrontal cortex. This network is consistent to the network reported in previous neuroimaging studies on episodic future imagination (Schacter et al., 2012), and very similar to the network activated for future imagination during DD studies (Peters and Büchel, 2010; Benoit et al., 2011). This analysis showed that the “core” network including the bilateral hippocampus is required in both the initial and later periods of the episodic future imagination.

During the initial period, more activation was found in the left middle temporal lobe extending to inferior frontal cortex and inferior parietal cortex, lower part of the left precuneus extending to the left hippocampus and fusiform gyrus, lower part of the left mPFC, as well as the right temporal and parietal areas. The fusiform gyrus has previously been shown to be more active during the initial construction period than the later elaboration period of both past and future events (Addis et al., 2007). The hippocampus is known to be the key region of past recollection and future imagination (Addis et al., 2011; Addis and Schacter, 2011); and has been previously shown to be more active in the initial construction period than the later elaboration period of future imagination (Addis et al., 2007). The increased MTL activation (fusiform gyrus, hippocampus) during the initial period may reflect higher level of memory search and retrieval process. Our results also revealed increased activity in the bilateral temporal lobes, bilateral inferior frontal cortices and left inferior parietal cortex during the initial period. These areas have been shown to be involved in various aspects of language processing, such as syntax (Makuuchi et al., 2009), cognitive control (Geranmayeh et al., 2014), pronunciation (Hu et al., 2013), as well as the emotional aspect (Wildgruber et al., 2004). It seems that areas typically associated with more complex language processing are involved during the initial period than the later period of future imagination. During the later period, more activation has been found in the left superior and middle frontal cortices, the upper part of the bilateral mPFC and the upper part of the bilateral precuneus. These results are in accordance with earlier observations of more activation during elaboration in a similar network (Addis et al., 2007; Ford et al., 2014) and may possibly reflect the self-referential and contextual processes associated with more elaborated imagination (Addis et al., 2007).

Using parametric analyses, previous studies have shown that brain regions such as ventral striatum, ACC/mPFC and PCC are responsive to subjective value of future reward (Kable and Glimcher, 2007; Peters and Büchel, 2009). The changes of the valuation signals in ACC/mPFC were found to be associated with the behavioral modulation effect (Peters and Büchel, 2010; Benoit et al., 2011). In our parametric analyses, we observed different patterns of subjective value signal changes during the initial and later periods of future imagination. During the initial period, SV signal changes were found in bilateral precuneus and middle cingulate cortices, right ACC, left superior middle frontal cortex and left insular with its adjacent superior temporal cortex. During the late period, SV signal changes were found in right ventral striatum and right hippocampus. All these areas have been revealed to be sensitive to absolute or subjective value of reward in previous studies (Kable and Glimcher, 2007; Ballard and Knutson, 2009; Peters and Büchel, 2009; Carter et al., 2010; Lebreton et al., 2013). Interestingly, the increased valuation signal in the right hippocampus was found only during the late period. This result is consistent with a previous study that revealed the hippocampus to play a critical role in the valuation of imagined future outcomes, and was associated with greater imagined detail (Lebreton et al., 2013). As mentioned earlier, the elaboration process involves the generation of additional details to fulfill the imagined events (Daselaar et al., 2008). It is possible that the vivid detail generated during a prolonged and more elaborated imagination may induce the changes of subjective value signal within the hippocampus.

In our experiment, it was not possible to ask subjects to indicate their specific time point of starting the elaboration process. Since the imagination task was imbedded into the decision making task, having a requirement for subjects to indicate the specific elaboration start time point would add to task complexity and most likely interfere with the decision making process. For our analyses, we separated our 8 s elaboration period into the first 3 s and the latter 5 s to closely resemble the initial period and later period of imagination, respectively. The choice of 3 s may seem arbitrary at first but was done so with the following understanding: (1) Usually, the construction of a completely new event takes about 7 s (Addis et al., 2007), however, we think the construction period of the majority of our subjects may have occurred within the first 3 s since a detailed interview was conducted right before the fMRI scans, and subjects were asked to re-construct the previously imagined event. It has been previously shown that the re-construction of a previously imagined event requires much less effort than the cognitive process of imagining a totally new event (Gaesser et al., 2013), and all of our subjects reported to be able to immediately retrieve the event when hearing the cue words. (2) It will be easier to compare our results with the results of previous study that used a 3 s stimuli presentation (i.e., Peters and Büchel, 2010). (3) We have previously discussed the differential roles of the left hippocampus and lateral cortical regions in the construction and elaboration process of future imagination. The time courses of the left hippocampus and left middle frontal cortex showed the peak voxels in either first 3 or later 5 s of time window, respectively. This indicated that our separation between the first 3 and later 5 s are reasonable. But future studies might help to better understand in more detail the specific timing.

To summarize this part, episodic future imagination evoked a “core” network including the hippocampus. More importantly, the current study shows different activation patterns to be associated with the initial and later period of future imagination. We suggest that those areas more associated with the initial period may support the memory search and retrieval process, and the complex language process, whereas those areas with stronger association to the later period may support contextual and self-referential processes that are especially prominent in the elaboration of future events. Parametric modulation analysis of the subjective value also showed a distinct pattern associated with both periods of future imagination. The SV signal changes in the hippocampus region may be related to increases in imagined details during the later period where subjects extensively elaborate their future events.

Our current study revealed correlations between the behavioral modulation effect and various neural mechanisms associated with either the initial or later period of future imagination. First, the neural responses to the initial period in the left anterior temporal pole were negatively associated with the behavioral modulation effect. The left anterior temporal pole has been suggested as a semantic hub for complex language function (Patterson et al., 2007; Tsapkini et al., 2011), and is inevitable for the processes such as retrieval of names of friends, loved ones and famous people (Abel et al., 2015). The negative association between the left anterior temporal activation and the behavioral modulation effect implied that semantic retrieval *per se* during the imagination task did not lead to a change of DD.

Second, the SV signal change in the right ACC and bilateral PCC during the initial period was significantly associated with the behavioral modulation effect. The association found for the right ACC replicates a previous study with a similar paradigm (Peters and Büchel, 2010). Moreover, the current study additionally showed the involvement of the bilateral PCC, which were also the key region for subjective valuation during intertemporal choices (Kable and Glimcher, 2007). Positive associations were also found between the behavioral modulation effect and the activation in left lateral prefrontal and parietal areas in response to the later imagination period. The lateral frontoparietal areas have been consistently associated with more future oriented decisions (McClure et al., 2004, 2007; Weber and Huettel, 2008; Figner et al., 2010; Christakou et al., 2011; Luo et al., 2012). In the context of intertemporal choices, the lateral frontoparietal regions have been associated with the deployment of cognitive control, promoting more “rational” decisions, and self-control over immediate gratification (Hare et al., 2009; Sellitto et al., 2011; Luo et al., 2012; Stoeckel et al., 2013).

The gPPI analyses showed a positive correlation between the behavioral modulation effect and the FC between the right ACC and the left hippocampus. This result reiterates the important role played by the hippocampus-mPFC pathway in the modulation of episodic future imagination (Peters and Büchel, 2010; Benoit et al., 2011). The positive association was also found between the behavioral modulation effect and the FC between the left IPC and the left hippocampus. No significant results were found when using left superior and middle cortices as seed regions for the PPI analyses. Animal studies have shown direct reciprocal connectivity between the IPC and the hippocampus and parahippocampus (Suzuki and Amaral, 1994; Rockland and Van Hoesen, 1999; Lavenex et al., 2002). In human, robust FC between the IPC and the hippocampus has been observed (Kahn et al., 2008; Wang et al., 2014), and the interaction between these two regions has been hypothesized to underpin the episodic memory retrieval process (Wagner et al., 2005). The hippocampal-cortical connectivity, including the hippocampal-IPC connectivity, has been suggested to underlie other aspects of cognition beyond memory functions to guide behavior (Ranganath and Ritchey, 2012). Our observed association between the behavioral modulation effect and the hippocampal-IPC connectivity provides additional evidence for the role of the cortical-hippocampal connectivity in episodic memory (imagination) guided behavior. Interestingly, the behavioral modulation effect was also correlated with the FC between the left IPC and the bilateral occipital lobes. This may be related to the vividness of imagined event.

The additional gPPI analyses revealed associations between the episodic memory capacity and afore-mentioned FC. These associations indicate that the neuronal processes underlying the behavioral modulation effect are related to episodic memory capacity. Both cortical areas (right ACC and left IPC) that are interacting with the hippocampus were thus thought to be important nodes to mediate the modulation effect of future imagination. Our hierarchical regression model further confirmed that BOLD signal changes in both the right ACC during the initial period, and in the left IPC during the later period, are important predictors of the behavioral modulation effect. Signal changes in the right ACC may be more related to change of the valuation process; while the signal changes in the left IPC maybe more related to the changes of the cognitive control process.

Whilst our results offer valuable insights into DD and the use of future imagination, this study has some limitations, which should be discussed and inform future research. First, our subjects may have slightly different time point for switching from the event construction to elaboration. Therefore, the separated two periods—the initial period of 3 s and the later period of 5 s, can only tentatively resemble the construction and elaboration period of imagination. In the current decision making paradigm, it is not appropriate to ask subjects to indicate the exact time point of switching by a button press. Future studies should be conducted to provide a more refined separation of the two periods. Second, we did not ask our subjects to rate the vividness of imagination during the fMRI task. Therefore, we were not able to make inference on the relationship between the vividness of imagination and increased FC between left IPC and occipital lobe, as well as with the degree of DD change. Finally, most of our subjects have a higher education level (i.e., university students). This sampling bias might have caused the ceiling effect on the measurement of the immediate and delayed recall of the VLMT test. Future studies should address the relationship between episodic memory capacity and the modulation effect of the future imagination in a more broader population.

In conclusion, the current study confirmed our hypothesis that episodic future imagination modulates intertemporal choices by reducing discount rates. Our results replicate the findings of earlier studies in that the hippocampal-mPFC pathway underlies the modulation effect. Using the paradigm of prolonged and more elaborated imagination, we also observed additional lateral prefrontoparietal areas to be related to the modulation effect. Among the lateral cortical regions, the left IPC was found to be an additional important node, since its interaction with the hippocampal area was both associated with the behavioral modulation effect and the episodic memory capacity. These results are consistent with the idea that multiple hippocampal-cortical pathways underlying the decision-making process (Buckner, 2010). And for the first time, our study demonstrates this underlying neuronal process to be associated with individual episodic memory capacity.

## Author contributions

XH, FJ, and BW conceived of and designed this study. XH, HK, DM, and MT conducted the study. XH, HK, JM, and YH contributed to the data analysis. XH, YH, FJ and BW contributed to the interpretation of the results. XH wrote the manuscript. JM, FJ, and BW critically revised the manuscript for important intellectual content. All authors approved the final version of this manuscript to be published and agreed to be accountable for all aspects of the work in ensuring that questions related to the accuracy or integrity of any part of the work be appropriately investigated and resolved.

## Funding

This research is supported by a research grant (JE 2707/7-1) from the German Research Council (DFG), Germany. BW was supported by a Heisenberg Grant (We 4427/3-2) from the DFG Germany.

### Conflict of interest statement

The authors declare that the research was conducted in the absence of any commercial or financial relationships that could be construed as a potential conflict of interest.
